# Speed and accuracy tradeoff in whole body movement during vertical jumps under varying landing constraints

**DOI:** 10.1038/s41598-025-04601-4

**Published:** 2025-06-06

**Authors:** Hiroki Murakami, Norimasa Yamada

**Affiliations:** 1 Kinjo University, Hakusan, Japan; 2 Chukyo University, Toyota, Japan

**Keywords:** Speed-accuracy trade-off, Whole-body motor control, Vertical jump, Entropy analysis, Motor control strategy, Feed-forward control, Human behaviour, Psychology

## Abstract

The speed-accuracy trade-off, described by Fitts’ law, has been well studied in fine motor tasks but remains insufficiently explored in whole-body movements, such as jumping. This gap limits our ability to identify universal motor control principles applicable to fine and gross motor tasks. To address this, we investigated the influence of landing accuracy constraints on vertical jump performance. Twelve participants performed jumps under four conditions: no accuracy constraints and progressively smaller target areas (100%, 65%, and 36% of the force-plate surface). Stricter accuracy demands a reduced jump height and systematic adjustments in the magnitude and direction of the take-off velocity. Notably, these trade-offs occurred despite the participants’ inability to continuously monitor the target during the jump, relying instead on the initial recognition of accuracy constraints. Entropy analysis revealed decreased variability in landing positions, reflecting precise motor adaptations to meet the task requirements. These findings suggest that principles similar to Fitts’ law govern speed-accuracy trade-offs in whole-body movements. This study provides valuable insights for sports, rehabilitation, and robotics applications by illustrating how accuracy constraints shape dynamic full-body movements.

**Supplementary Information:**

The online version contains supplementary material available at 10.1038/s41598-025-04601-4.

## Introduction

The relationship between speed and accuracy fundamentally governs human movements, often called the speed-accuracy trade-off^1^. Based on Woodworth’s experimental^2^ results on hand movements, Fitts^3^ developed a strong relationship between speed and accuracy in experiments involving repetitive hand movements using a stylus. Fitts demonstrated that movement time (MT) increases linearly with task difficulty (ID), where a higher ID implies greater accuracy. Additionally, because an increase in ID indirectly implies a demand for higher accuracy, the relationship (speed and accuracy trade-off) can be read from the experimental results as MT increases (average speed decreases) as the demand for accuracy increases. Furthermore, this trade-off, encapsulated in Fitts’ law, connects motor control to information theory, borrowing concepts from Shannon’s^4^ framework. Fitts’ experimental results, also called “Fitts’ law,” are recognized as a vital law describing human physical movement’s characteristics^5^. Moreover, pointing devices have become widely used with the development of computers. It is necessary to evaluate and predict their performance before selecting and designing input devices and interfaces, to which Fitts’ law has contributed^6–8^. The application of Fitts’ law to human-computer interaction (HCI) has significantly influenced the design and evaluation of devices such as PCs and tablets^9^. Furthermore, although the movements that led to this law are simple and repetitive hand movements, various movements have been extended to apply to this law. For example, movements using a joystick or a mouse are applicable^10,11^. Studies are also underway to determine whether this law holds for sports movements that require full-body movements.

However, whether the speed–accuracy trade-off described by Fitts’ law can be generalized to whole-body movements remains an open question^9^. Several studies have supported the trade-off in full-body sports actions, such as dart throwing, soccer kicking, and cricket throwing^12–14^. Nonetheless, other studies have shown that increasing movement time does not necessarily improve accuracy, as seen in golf putting and fencing^15–17^. These inconsistencies raise critical questions: When and how does the speed-accuracy trade-off emerge in whole-body movements? What mechanisms underlie this relationship? This may be because the movements in many competitive sports are complex and involve multi-joint movements, making it challenging to identify the factors involved^9^.

In our previous research^9^we explored how introducing a landing position control task to a vertical jump could highlight the speed-accuracy trade-off in whole-body movements. This study suggested that altering movement strategies, including vertical velocity control during take-off, could improve accuracy. Further, we built on these findings and applied an entropy-based trajectory data analysis to assess movement variability and information processing demands. Details of the previous study’s entropy computation and experimental outcomes are described in the Methods and Discussion sections, respectively. In our previous research^9^this analysis revealed that instructing participants to land on the take-off point reduced entropy in the landing positions, indicating increased movement precision under verbal instructions alone.

While previous research is also fascinating in that it showed the complexity of human movement even when the landing position was restricted by verbal instruction alone, in an environment where actual sports movements occur, there are cases where it is necessary to achieve a high level of accuracy within the constraints of the physical space. For example, in the long track and field jump, the athlete must match their foot with the take-off board without losing any speed gained in the approach run. Specifically, it is essential to clarify how much speed changes when accuracy is required owing to physical constraints. Therefore, by examining the height of the jump when the level of accuracy needed is changed by physically restricting the landing position in the vertical jump, the relationship between speed and accuracy and the mechanisms involved in sports movements that require whole-body movement can be further clarified.

We manipulated landing accuracy demands by varying the size of the permissible landing zone in a vertical jump task. The participants performed jumps under four conditions ranging from no constraint to narrow target zones, and motion data such as jump height, 3D velocity vectors, and entropy were analyzed. We hypothesized that greater accuracy demands would lead to a lower jump height and reduced velocity at take-off, indicating a shift in the motor control strategy.

## Methods

### Participants

The participants were 12 men (21.7 ± 3.5 age, height: 175 ± 6.2 cm, weight: 72.3 ± 10.7 kg) and older, belonging to an athletic club. The participants were not recruited based on specific athletic disciplines. Instead, this study investigated fundamental motor control strategies under different accuracy constraints in whole-body movements. Therefore, athletes from various sports backgrounds (e.g., soccer and volleyball) who were interested in the experimental purpose voluntarily participated. The sample size was determined based on our previous study^9^which reported the significant effects of landing accuracy constraints in a similar vertical jump paradigm. Post-hoc power analysis using G*Power (version 3.1) indicated that with 12 participants, the current study had a statistical power greater than 0.80 to detect a medium-to-large effect size (*f* = 0.25, *α* = 0.05, repeated measures ANOVA, four conditions). The repeated-measures design and use of multiple trials per condition further enhanced the sensitivity and statistical robustness of the analysis. All study procedures were conducted per the Declaration of Helsinki and the ethics code of Chukyo University and were approved by the ethics committee of Chukyo University (approval number: 2023-088). All participants provided written informed consent before participation.

### Apparatus

Signals from a single force plate (Bertec Corporation, Columbus, OH, USA) digitized at 1000 Hz (force data in three axial directions (x, y, z) and the center of pressure (cop) in two axial directions (x, z)) were imported onto a PC.

## Experimental design

**Fig. 1:**
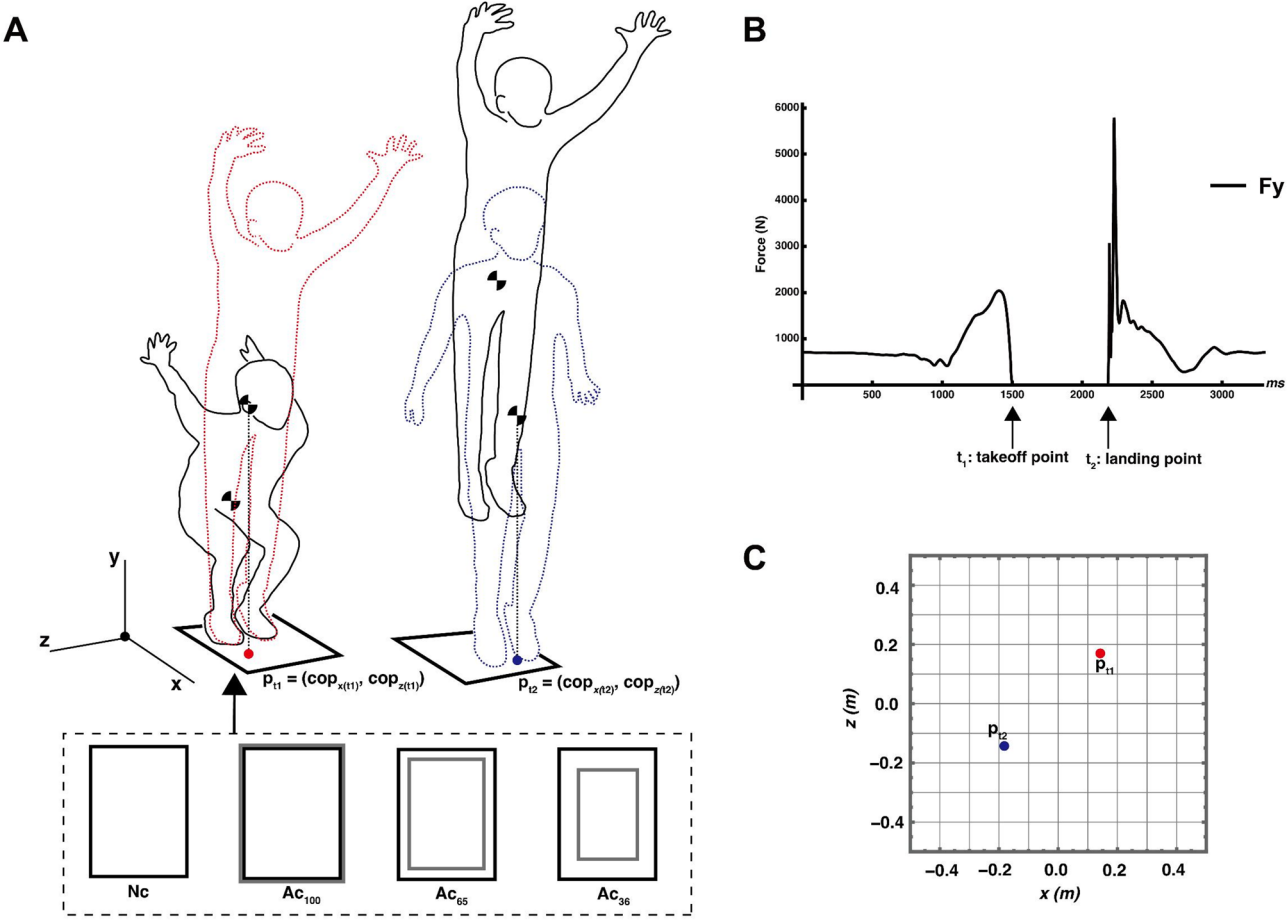
Conceptual diagram of the experimental setup, condition, and primary data obtained from the force plate. (Adapted from Fig. 1 in Murakami & Yamada (2024)). (**A**) An example of a vertical jump trial illustrating the phases: sinking to take-off (black solid line transitioning to red wavy line) and aerial phase to landing (black solid line transitioning to blue wavy line). The coordinates of the take-off point (p_t1_) and landing point (p_t2_) were computed from the center of pressure (COP) data. Four conditions were tested: one with no constraints on landing accuracy (Nc) and three adjusted conditions (Ac_100_, Ac_65_, Ac_36_) with physically constrained landing areas of varying sizes. The landing areas were defined as the white regions inside a black border (3 cm wide) on sheets placed on the force plate. These areas corresponded to the following sizes: Ac_100_: 100% of the force plate’s surface area (60 × 40 cm^2^), Ac_65_: 80% of the force plate’s surface area (48 × 32 cm^2^), Ac_36_: 60% of the force plate’s surface area (36 × 24 cm^2^). (**B**) A typical example of vertical ground reaction force data (Fy) from the force plate. The force value is zero during the aerial phase (when not in contact with the force plate). (**C**) A typical example of x-z plane coordinates of the take-off and landing points. The origin (p_0_) represents the initial value (at rest).

A conceptual diagram of the experiment is shown in Fig. 1. After a sufficient warm-up, the participants performed vertical jumps on a force plate under the following four conditions:

1. Normal condition (Nc): They were instructed to jump with maximum effort without specific landing constraints.
2. Adjusted conditions: The participants were instructed to focus on landing within a designated area. The landing area was the white region inside, bordered by a 3 cm black line on a sheet placed on the force plate. Preliminary tests were conducted to assess the feasibility of different target areas in determining the adapted landing accuracy conditions. It was found that landing zones narrower than 36% of the force plate area forced the participants to adopt an unnaturally narrow stance from the beginning of the trial. In this task, once the participants stepped onto the force plate, their foot placement, including the stance area, was fixed as the starting position and remained unchanged until take-off. Thus, excessively narrow target areas impose physical constraints on the initial stance rather than landing control. These constraints introduced confounding effects on jump performance unrelated to the study objective. Therefore, the narrowest condition was set to 36%, and incremental increases in the target area relative to the baseline determined the remaining adapted conditions. The landing area sizes were as follows:
3. Ac_100_: Covered 100% of the force plate’s surface (60 × 40 cm²).
4. Ac_65_: Covered 65% of the force plate surface by reducing the long and short sides to 80% of their original length (48 × 32 cm²).
5. Ac_36_: Covered 36% of the force plate surface by reducing the long and short sides to 60% of their original length (36 × 24 cm²).

Before each trial, they were allowed to inspect the landing area but were instructed not to look at their feet while jumping, similar to normal conditions. The participants were instructed to jump with maximum effort in all the trials. They were given sufficient rest between trials and were allowed to take additional breaks upon request.

## Experimental procedure

Participants entered the laboratory and were briefed on the experimental tasks and potential risks. They were given 10 min for a warm-up. Before the warm-up, it was announced that the experimental task involved vertical jumping; however, specific experimental conditions were not disclosed—first, the participants performed five normal condition (Nc) trials. Subsequently, three adjusted conditions (Ac_100_, Ac_65_, and Ac_36_) were performed randomly, with five trials for each condition. All adjusted trials were presented in a fully randomized order across the four conditions to minimize potential learning or order effects. The participants did not consecutively complete all five trials for a given condition but performed trials from different conditions in a randomized sequence. Additionally, the trials were not repeated even if the participants landed outside the designated area. The landing zone was visually presented before each trial, and the participants were instructed to attempt to land within the specified region. However, whether they succeeded was not the focus of this study and was not used as a criterion for excluding or repeating the trials. Corrective feedback was not provided during the experiment. While the participants may have gradually developed a sense of the target zone locations over time, the randomized trial presentation and limited number of repetitions helped minimize such effects. No feedback regarding their performance was provided until all trials were completed.

### Data analysis

All numerical calculations, including the analyses, were performed using Mathematica 13.0.1.0 (Wolfram Research, Champaign, IL, USA).

### Calculation of acceleration, velocity, and position vectors of the center of gravity

All data collected from the force plate (FP4060-07-TM-2000, Bertec, USA, Natural Frequency: Fx: 300, Fy: 300, Fz: 450) were raw, and no smoothing was applied. No smoothing filtering was applied to the force signals to retain the natural variability of the data, which is essential for entropy analysis and other variability-sensitive computations. This decision was made to avoid attenuating the signal fluctuations that may reflect meaningful aspects of motor control. To verify the validity of this approach, we conducted a frequency analysis using a Fast Fourier Transform (FFT) and Power Spectral Density (PSD) on unfiltered force signals. The analysis was limited to the portion of data from the start of the movement to take-off (Supplementary Fig. 1A). Supplementary Figs. 1B show that most spectral power was concentrated below 10 Hz across all three axes (Fx, Fy, and Fz). The PSD values, plotted in physical units (N²/Hz), demonstrated a rapid decline in power beyond this range, indicating a minimal effect of high-frequency noise. The DC offset (0 Hz) was excluded from the PSD to avoid bias from the constant components. Therefore, no filtering was applied to preserve significant signal variability relevant to the entropy-based analysis.

Before calculating each performance variable, the following variables were calculated from the force vector $\:{\varvec{F}}_{t}$ obtained from the force plate. $\:{\varvec{F}}_{t}$ is the sum of the temporal variation of the body’s movement $\:m{v}_{t}$, gravitational acceleration vector g and weight multiplied by the mass m of the experimental participants and is expressed according to Eq. 1 as follows:

1 $$\:\begin{array}{c}{\varvec{F}}_{\varvec{t}}=m\frac{d{\varvec{v}}_{\varvec{t}}}{dt}+mg,\:g=\left(0,\:9.81,\:0\right).\:\end{array}$$.

From Eq. 2, the acceleration vector $\:{a}_{t}$ of the center of gravity becomes:

2 $$\:\begin{array}{c}{\varvec{a}}_{\varvec{t}}=\frac{d{\varvec{v}}_{\varvec{t}}}{dt}=\frac{{\varvec{F}}_{\varvec{t}}}{m}-g.\:\end{array}$$.

Therefore, if the time when the movement starts is 0 and the time when the foot leaves the force plate is $\:{t}_{1}$ (take-off time), the experiment starts from a stationary state (the initial value of the velocity vector is 0), and the initial value of the center of gravity position at rest coincides with the center of pressure ($\:COP$), which is obtained from the force plate, the following equation is used to calculate the movement speed. The velocity ($\:{v}_{t}$) and position ($\:{p}_{t}$) vectors from the start of the movement to take-off time were calculated using the following equations:

3 $$\:\begin{array}{c}{\varvec{v}}_{\varvec{t}}={\int}_{0}^{{t}_{1}}{\varvec{a}}_{\varvec{t}}dt+{\varvec{v}}_{0},\:{\varvec{v}}_{0}=(0,\:0,\:0). \end{array}$$

4 $$\:\begin{array}{c}{\varvec{p}}_{\varvec{t}}={\int\:}_{0}^{{t}_{1}}{\varvec{v}}_{\varvec{t}}dt+{\varvec{p}}_{0},\:{\varvec{p}}_{0}=\left({cop}_{x\left(0\right)},\:0,\:{cop}_{z\left(0\right)}\right).\:\end{array}$$.

**Fig. 2:**
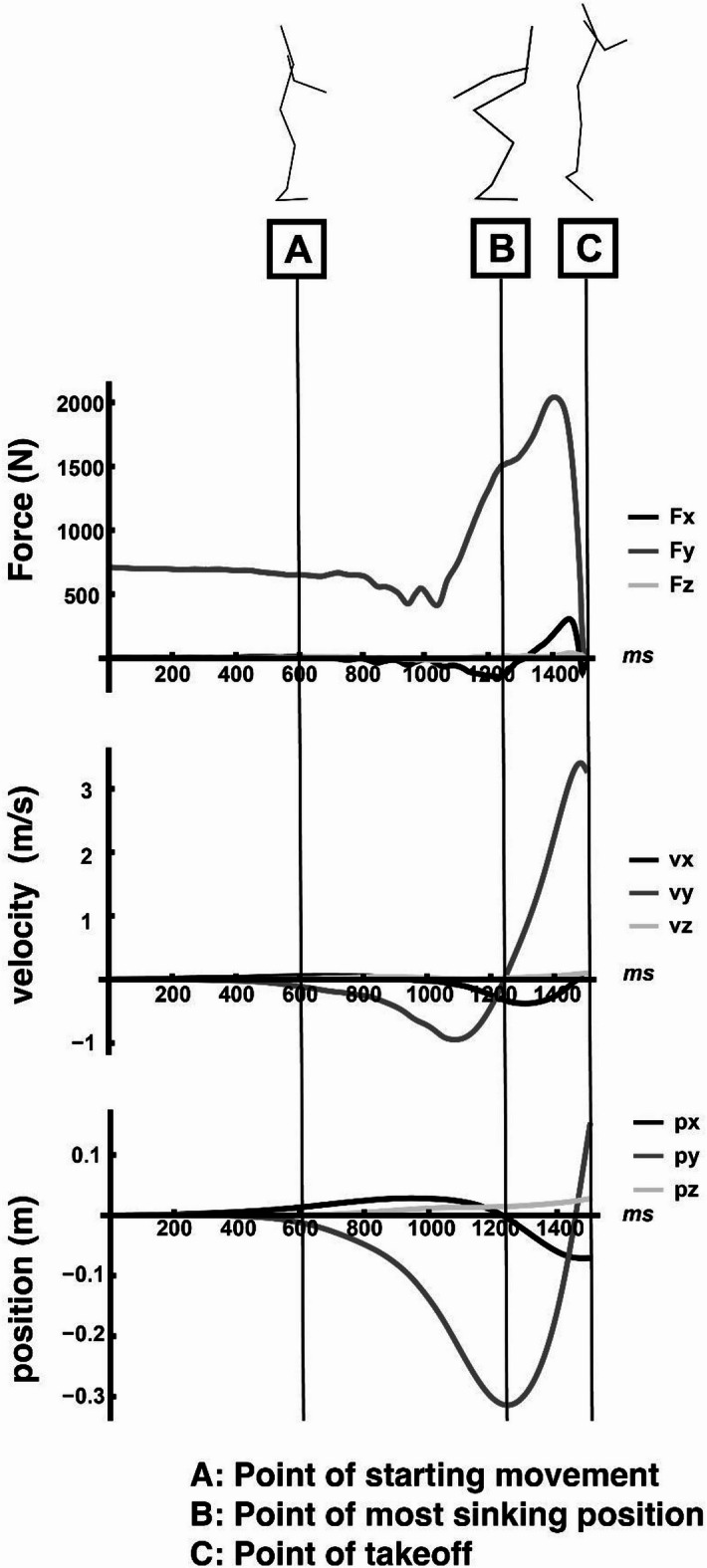
A typical example of analyzed data (above: each component of 3D force, middle: each component of 3D velocity, below: each component of 3D position). (Adapted from Fig. 2 in Murakami & Yamada (2024)). Typical examples of each variable used in the analysis: The starting movement point (**A**, at ± 10% of the resting position), most vertical sink point (**B**, t_0_), and take-off point (**C**, t_1_) were calculated as the characteristic points common to all trials.

The trapezoidal rule was used for all numerical integrations. A typical example of the time-series data for the acceleration, velocity, and position vectors in each axis direction is shown in Fig. 2.

### Position of the center of pressure at take-off and landing

The take-off and landing time points coincided with the falling and rising points in the force data (Figs. 1B, $\:{\text{t}}_{1}$, and $\:{\text{t}}_{2}$). These values are the coordinates of the xz plane of the COP in each trial but were converted to coordinates by subtracting the initial value ($\:{p}_{0}$) from the following equation. As gravity is the only force acting on the center of gravity during the dwell time, the center of gravity during the dwell time is in constant acceleration movement in the y-axis direction and constant velocity movement in the x- and z-axis directions (between the red and blue wave lines in Fig. 1A).

5 $$\:\begin{array}{c}{p}_{t1}=\left({cop}_{x\left(t1\right)},\:{cop}_{z\left(t1\right)}\right)-\:\left({cop}_{x\left(0\right)},\:{cop}_{z\left(0\right)}\right).\end{array}$$

6 $$\:\begin{array}{c}{p}_{t2}=\left({cop}_{x\left(t2\right)},\:{cop}_{z\left(t2\right)}\right)-\:\left({cop}_{x\left(0\right)},\:{cop}_{z\left(0\right)}\right).\end{array}$$.

### Distance between take-off and landing point

The distance between the two points ($\:{p}_{t1}$ and $\:{p}_{t2}$) was calculated in the xz plane. This variable is important for examining whether the landing accuracy changes depending on the conditions. The movement is adjusted according to the required accuracy (conditions) if the value is small. According to previous research^18^this variable can be examined with jumping height to determine the trade-off relationship between speed and accuracy.

### Jump height

The jumping height was calculated according to the following equation using the y-axis velocity at take-off: This value corresponds to the maximum value in the y-axis direction in Eq. 7.

7 $$\:\begin{array}{c}h=\frac{{{v}^{2}}_{y\left({t}_{1}\right)}}{2{g}_{y}}.\:\end{array}$$.

Although take-off velocity is used in calculating jump height and represents a more direct measure of movement speed, we adopted jump height as the primary performance indicator. This choice was made to maintain consistency with our previous research^9^ on whole-body movements under accuracy constraints. Jump height is a functionally relevant and interpretable outcome variable commonly used in applied motor control and sports science research.

### Quantification of each point by information entropy

To quantify movement variability and estimate information processing demands, we calculated the entropy of 3D movement trajectories based on the method described in our previous study^9^. This analysis is based on Shannon’s information theory^4^where entropy is computed from the probability distribution of coordinates at specific time points during the trajectory (e.g., the midpoint and endpoint). A decrease in entropy from one point to another in the direction of time indicates reduced irregularity and is interpreted as an increase in information processing. This technique allows a detailed temporal assessment of the accuracy achieved through movement modulation. Therefore, this temporal comparison provides insight into when movement precision emerges during jumps. Using this analytical approach, kinematic research can compare multiple movement trajectories using entropy to understand better when and how information processing is required to improve accuracy and performance.

Using the x-z plane coordinates of the position vector at take-off and landing for each trial and the coordinates of the 3D velocity vector at take-off, entropy was calculated as an index for evaluating the variability of the vertical jump^9^. All variables included all the coordinates for each condition, and entropy was calculated using methods described in previous studies^9,19,20^. Each coordinate at each time point was encoded into a square or cube (0.1 m per side, and entropy was calculated by computing the probabilities^9,19,20^. This study analyzed the center of gravity trajectory of whole-body movement. Therefore, to ensure the accuracy of the analysis, as in previous studies that targeted hand movement^19,20^we set the bin width per the method used in an earlier study^9^. This entropy was calculated using $\:{\varvec{H}}_{1}\left(\varvec{X}\right)\equiv\:\underset{\varvec{a}\to\:1}{\mathbf{lim}}{\varvec{H}}_{\varvec{a}}\left(\varvec{X}\right)=\sum\:{\varvec{P}}_{\varvec{i}}{\mathbf{log}}_{2}(1\:/\:{\varvec{P}}_{\varvec{i}})$, where $\:{\varvec{P}}_{\varvec{i}}$ is the frequency distribution of data points in bin *i*. The limiting value of $\:{\varvec{H}}_{\varvec{a}}$ as a→1 is Shannon entropy^4,21^. In other words, in this analysis, if all coordinates are in the same square or cube part (encoded with the same value), the value of the information entropy is zero^9,19,20^.

### Velocity vector angle of deviation from the vertical direction

Calculate the angle of deviation from the most vertical sink point ($\:{t}_{0}\text{i}\text{n}\:\text{F}\text{i}\text{g}\text{u}\text{r}\text{e}\:2$) to the take-off point ($\:{t}_{1}\text{i}\text{n}\:\text{F}\text{i}\text{g}\text{u}\text{r}\text{e}\:2$), which is the inner product of the ***v*** and ***k*** vectors (Eqs. 8, ^9^).

8 $$\:\begin{array}{c}{v}_{{ang}_{\left(t\right)}}={\text{cos}}^{-1}\frac{{\varvec{v}}_{\varvec{t}}\cdot\:\varvec{k}}{\parallel {\varvec{v}}_{\varvec{t}}\parallel},\:k=\left(0,\:1,\:0\right),\:\left(t={t}_{0}\sim{t}_{1}\right).\:\end{array}$$.

### Duration movement time of movement phase

Following the previous study^9^we calculated two duration movement times: the duration movement time from the starting movement point (at ± 10% of the resting position) to the time to the most vertical sink point ($\:{t}_{0}$), and the duration of movement time from the time to the most vertical sink point ($\:{t}_{0}$) to the take-off point ($\:{t}_{1}$).

### Statistical analysis

The primary objective of this study was to examine trends across trials rather than individual tendencies. To achieve this, we analyzed all trial data (12 participants × 5 trials per condition = 60 trials per condition), increasing the findings’ statistical power and precision. Treating each trial as an independent observation was justified by the randomized order of the trials and the provision of sufficient rest between jumps to minimize intertrial dependencies. We conducted a secondary analysis based on participant-level means using repeated-measures ANOVA to validate the findings’ robustness. This yielded consistent results, confirming the reliability of the observed effects. Instead, the design ensured that each trial was conducted independently with sufficient rest between jumps and randomized order of conditions, minimizing dependencies between trials and allowing them to be treated as independent observations. The average of five trials for each participant under Nc was set to 1, and the jump heights were expressed as proportions relative to this value to standardize individual differences in jump performance. The results for jump height, distance, 3D velocity vector scalar, and angle of deviation from the vertical direction are shown as mean values and standard deviations for all participants. Linear mixed-effects models (LMMs) were used to analyze the data, and relevant statistical assumptions were verified before model fitting. Specifically, the normality of residuals was confirmed using the Shapiro–Wilk test and the homogeneity of variance across conditions was assessed using the Levene test. Additionally, the independence of observations was ensured by randomizing the order of the conditions and including sufficient rest between trials to minimize fatigue effects. In the LMMs, Condition (Nc, Ac_36_, Ac_65_, Ac_100_) was treated as a fixed effect, with the control as the reference level, and the participant was included as a random intercept to account for repeated measurements. Model outcomes regarding estimated coefficients (β), standard errors (SE), and corresponding p-values were reported. As part of our planned analysis, we performed pairwise comparisons between adjacent accuracy conditions (Ac_36_ vs. Ac_65_ and Ac_65_ vs. Ac_100_) using the Bonferroni correction to account for multiple comparisons. The adjusted p-values are explicitly reported in the Results section to enhance clarity. Entropy, which quantifies the variability in the landing position, was calculated using the two-dimensional (x-z plane) position vector at the landing point. This analysis provided a measure of movement consistency and accuracy across conditions. Mathematica (Version 13.0.1.0; Wolfram Research, IL, United States) was used for statistical analysis, with a significance level of *p* < .05.

Linear mixed-effects models (LMMs) were employed to analyze the data for all outcome variables. LMMs are particularly well suited for repeated-measures data structures where the assumption of independence is violated, such as in the present design, where each participant completed multiple trials under each condition. In the models, the condition was treated as a fixed effect, and participants were included as a random intercept to account for the within-participant correlation of repeated observations. This approach allows the retention of trial-level data while appropriately accounting for the nested structure of the data^22,23^. Recent research has recommended this modelling strategy to improve statistical precision and control for Type I errors in designs involving repeated observations per participant^24^. Furthermore, LMMs enable robust parameter estimation, even when the number of observations per participant varies or when random effects need to be modeled flexibly. We conducted a secondary analysis based on participant-level means using repeated-measures one-way ANOVA to validate the robustness of the findings. The results are consistent for both methods, confirming the reliability of the observed effects.

## Results

### Jump height of the vertical jump

**Fig. 3:**
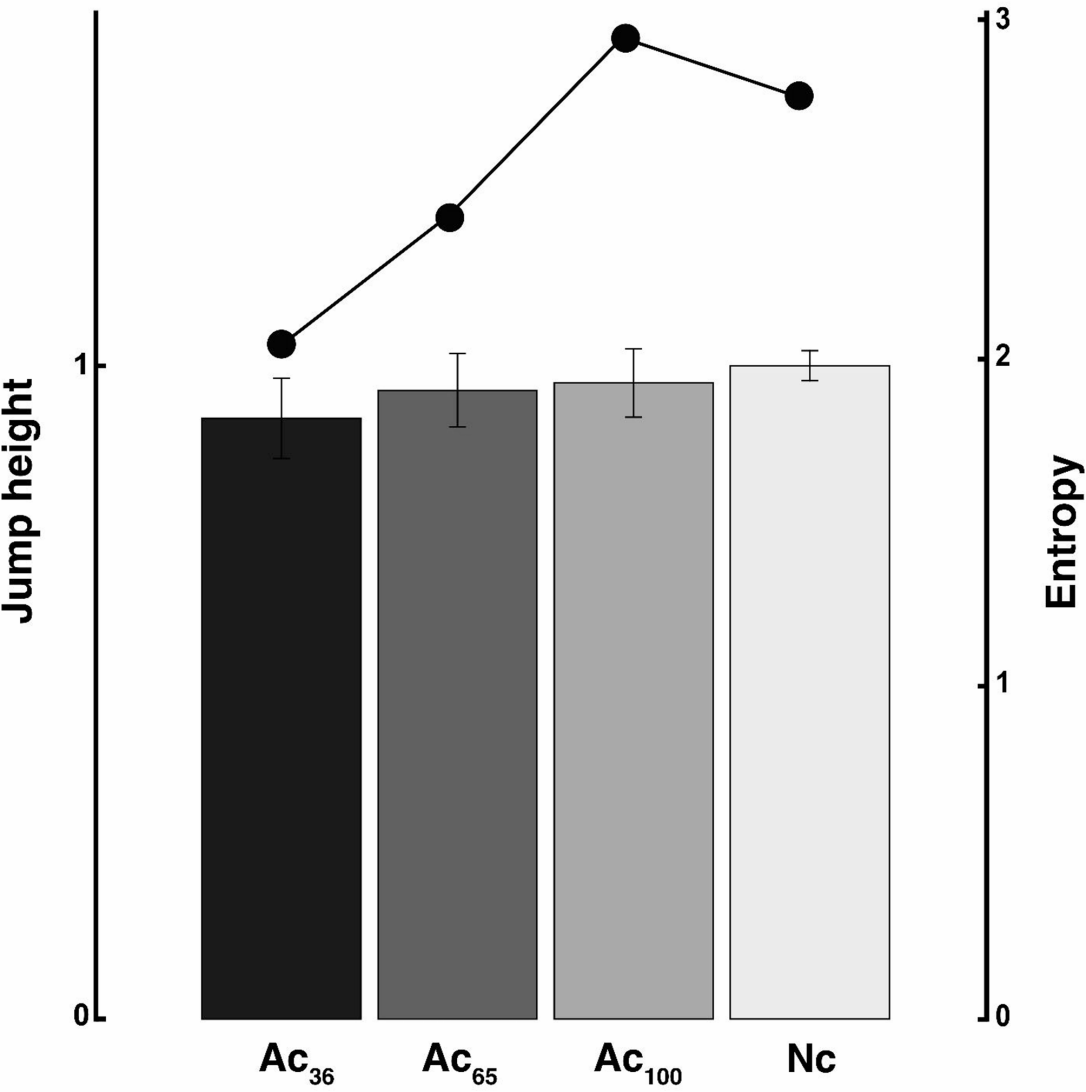
Jump height and entropy of landing position for each condition. The bar graph shows the jumping height, and the line graph shows the landing point entropy.

Jump height significantly decreased as the required landing accuracy increased (Fig. 3, Ac_36_:0.92 ± 0.06, Ac_65_:0.96 ± 0.06, Ac_100_:0.97 ± 0.05, Nc: 1.00 ± 0.02). Linear mixed-effects models (LMMs) revealed significant differences in jump height across the four conditions, with Nc set as the reference level. Compared to the Nc, jump height was significantly lower in Ac_100_ (*β* = -0.026, SE = 0.007, *p* < .001), Ac_65_ (*β* = -0.038, SE = 0.007, *p* < .001), and Ac_36_ (*β* = -0.080, SE = 0.007, *p* < .001). The pairwise comparisons using the Bonferroni correction revealed that the difference in jump height between Ac100 and Ac65 was statistically significant (*p* = .050). Further, the difference between Ac_65_ and Ac_36_ was highly significant (*p* < .001). Although the difference between Ac_100_ and Ac_65_ was marginal, these results suggest a graded modulation of jump performance across increasing accuracy demands. For validation, a secondary analysis using repeated-measures ANOVA based on participant-level averages for each condition (*F* [3, 16] = 30.73) yielded consistent results with generally high effect sizes (e.g., *η*² = 0.71^25^) and statistical significance (e.g., *p* < .001). This confirmed the robustness of these findings. As described in Eq. 7, jump height is determined by the vertical take-off velocity: Accordingly, the LMM analysis for y-axis velocity at take-off also showed a significant effect of condition: Compared to Nc (2.72 ± 0.24 m/s), velocity was significantly lower in Ac_36_ (2.58 ± 0.29 m/s, *β* = -0.142, SE = 0.034, *p* < .001) and Ac_65_ (2.62 ± 0.27 m/s, *β* = -0.102, SE = 0.034, *p* = .003), while the difference between Ac_100_ (2.67 ± 0.22 m/s) and Nc (*β* = -0.067, SE = 0.034, *p* = .050) was marginal. Post-hoc comparisons of adjacent conditions (Ac_36_ vs. Ac_65_ and Ac_65_ vs. Ac_100_) further supported a gradual reduction in take-off speed under increased accuracy constraints (*ps* < 0.05).

### Accuracy of vertical jump by distance and entropy

**Fig. 4:**
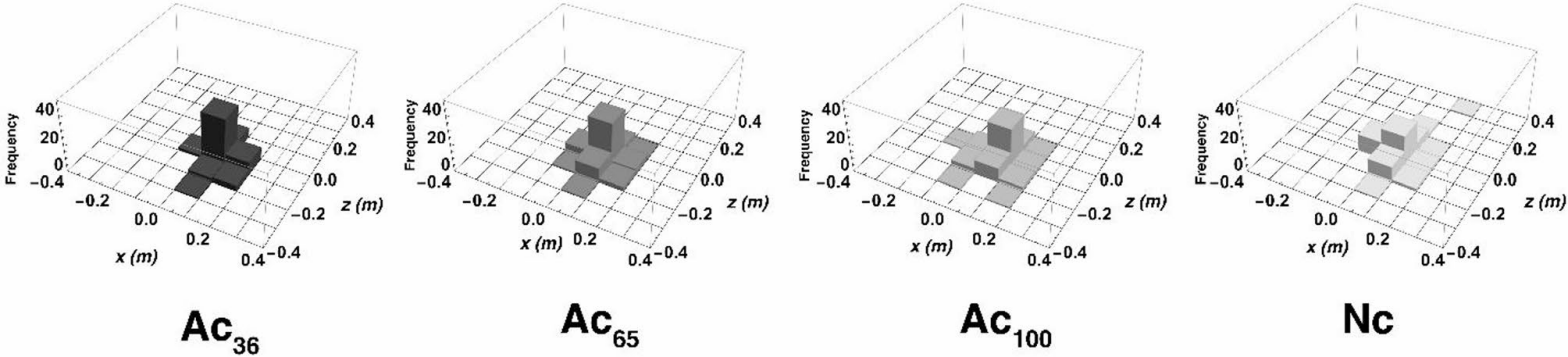
3D histogram of cop-coordinates of relative landing point for each condition. The 3D histogram shows the number of coordinates (x-z plate) corresponding to each grid required for the entropy calculation for each of the four conditions. Mean distances between take-off and landing positions (in the xz plane) were as follows: Ac₃₆: 0.17 ± 0.01 m, Ac₆₅: 0.20 ± 0.01 m, Ac₁₀₀: 0.24 ± 0.02 m, Nc: 0.21 ± 0.02 m. These values reflect the spatial deviation in the landing position, indicating changes in movement accuracy across conditions. Figure was created using Mathematica version 13.0.1.0 (Wolfram Research Inc., https://www.wolfram.com/mathematica/).

Figure 4 shows a 3D histogram of the 2D cop coordinates (x-z plane) of the relative landing point. To quantify the variation in these coordinates, we calculated the entropy and distance between the coordinates of the two points (take-off and landing) using the method described in previous studies^9,19,20^.

The distance between the take-off and landing points was analyzed using a linear mixed-effects model (LMM), with the condition as a fixed effect and the participant as a random intercept. Nc was used as the reference level. Compared to Nc (0.21 ± 0.02 m), distance was significantly shorter in Ac_36_ (0.17 ± 0.01 m, *β* = -0.047, SE = 0. 010, *p* < .001) and Ac_65_ (0.20 ± 0.01 m, *β* = -0.019, SE = 0.010, *p* = .050), while distance in Ac_100_ was significantly longer than Nc (0.24 ± 0.02 m, *β* = +0.030, SE = 0.010, *p* = .002). Post hoc comparisons on adjacent accuracy levels showed significant differences between Ac_36_ vs. Ac_65_ and Ac_65_ vs. Ac_100_ (*ps* < 0.05).

The relative entropy of the coordinates of the landing point, converted to values with the coordinates of the take-off point as the origin, decreased as the conditions requiring greater accuracy increased, and Nc was smaller than Ac_100_ as the distance (Fig. 3, Ac_36_:1.99, Ac_65_: 2.40, Ac_100_: 2.94, Nc: 2.77). This suggests that increased information processing during the aerial phase, as indicated by reduced landing entropy, reflects anticipatory motor planning before takeoff rather than real-time adjustments made during jumps.

### Scalar amount and angle of deviation from the vertical of the 3D velocity vector

**Fig. 5:**
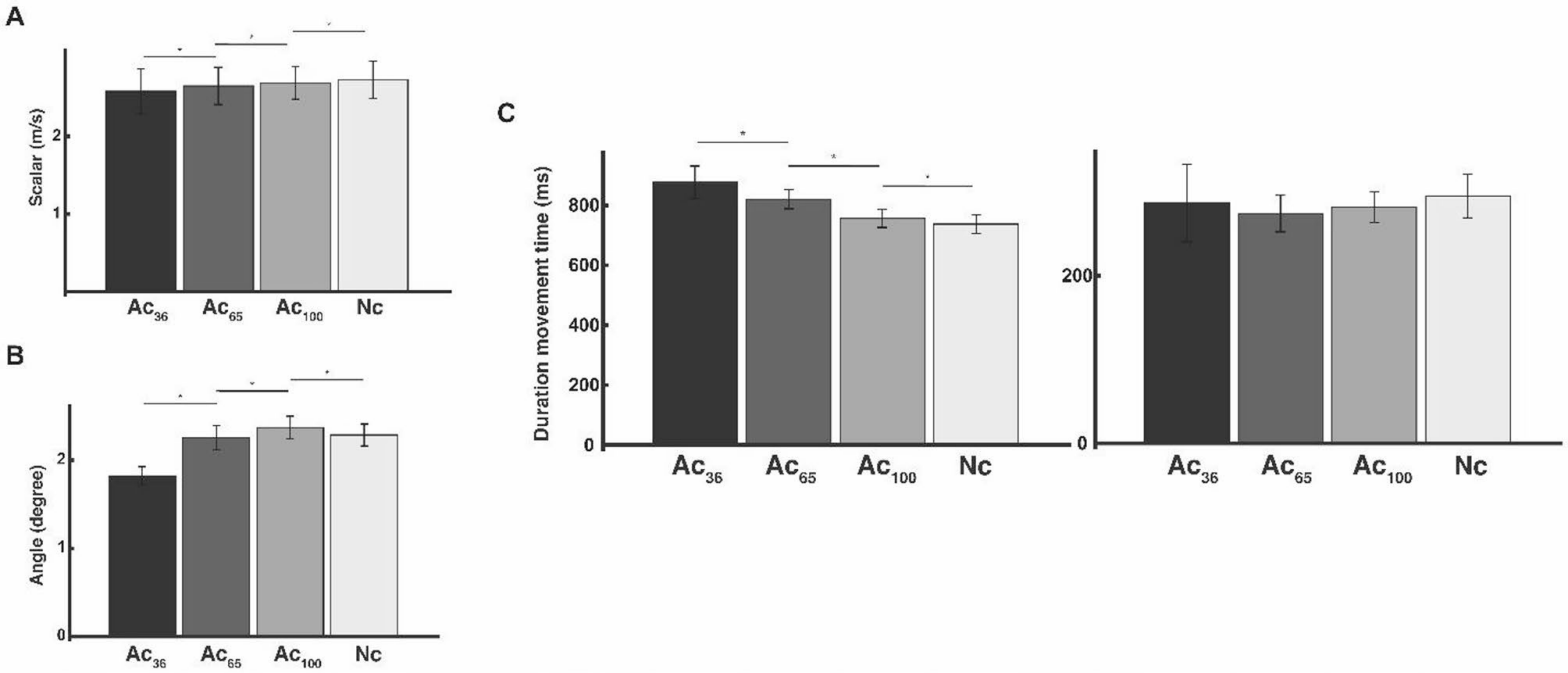
The performance variables (scalar, angle, and duration movement time of movement phase) for each condition. (**A**) The mean scalar of the 3D velocity vector at the take-off point for each condition. (**B**) The mean angle of deviation from the vertical direction at the take-off point for each condition. (**C**) The duration movement time of the starting movement point to the most vertical sink point (left) and the most vertical sink point to the take-off point (right). * Significant at *p* < .05.

The mean scalar of the 3D velocity vector for all participants at the take-off point is shown in Fig. 5A (Ac_36_:2.58 ± 0.29 m/s, Ac_65_:2.65 ± 0.24 m/s, Ac_100_:2.69 ± 0.21 m/s, Nc: 2.73 ± 0.24 m/s). Linear mixed-effects models (LMMs) revealed significant differences in the mean scalar of the 3D velocity vector across the four conditions, with Nc set as the reference level. Compared to the Nc, those scalar ware significantly lower in Ac_100_ (*β* = -0.047, SE = 0.033, *p* = .050), Ac_65_ (*β* = -0.087, SE = 0.033, *p* = .009), and Ac_36_ (*β* = -0.157, SE = 0.033, *p* < .001). Post hoc comparisons focused on adjacent accuracy levels showed significant differences between Ac_36_ vs. Ac_65_ and Ac_65_ vs. Ac_100_, indicating graded modulation of scalars in response to increasing accuracy demands (*ps* < 0.05). Regarding the angle of deviation from the vertical, the linear mixed-effects models (LMMs) showed a significant effect on the condition, with Nc set as the reference level (Fig. 5B). Compared to the Nc (2.30 ± 0.97-degree), the angle of deviation was significantly larger in Ac_100_ (2.37 ± 1.00-degree, *β* = 0.072, SE = 0.172, *p* = .049), and significantly smaller in Ac_65_ (2.26 ± 1.09-degree, *β* = -0.047, SE = 0.172, *p* = .050) and Ac_36_ (1.82 ± 0.80-degree, *β* = -0.487, SE = 0.172, *p* = .005). Post hoc comparisons focused on adjacent accuracy levels showed significant differences between Ac_36_ vs. Ac_65_ and Ac_65_ vs. Ac_100_, indicating graded modulation of scalars in response to increasing accuracy demands (*ps* < 0.05).

### Duration movement time of movement phase

The duration movement time of the starting movement point to the most sinking point is shown in Fig. 5C (Ac_36_:876 ± 19 ms, Ac_65_:820 ± 15 ms, Ac_100_:756 ± 15 ms, Nc: 737 ± 15 m/s). Linear mixed-effects models (LMMs) revealed significant differences in movement time across the four conditions, with Nc set as the reference level. Compared to the Nc, those duration movement time ware significantly longer in Ac_100_ (*β* = +19 ms, SE = 2.333, *p* < .001), Ac_65_ (*β* = +83 ms, SE = 2.333, *p* < .001), and Ac_36_ (*β* = +139 ms, SE = 2.333, *p* < .001). Post hoc comparisons focused on adjacent accuracy levels showed significant differences between Ac_36_ vs. Ac_65_ and Ac_65_ vs. Ac_100_, indicating graded modulation of scalars in response to increasing accuracy demands (*ps* < 0.001). A linear mixed-effects model revealed no significant effect of the condition on the movement duration from the most vertical sink point to the take-off point. Compared to the Nc (295 ± 10 ms), durations were slightly shorter in Ac_36_ (287 ± 13 ms, *β* = -8.4 ms, SE = 16.133, *p* = .603), Ac_65_ (274 ± 9 ms, *β* = -21.0 ms, SE = 16.133, *p* = .197), and Ac_100_ (282 ± 8 ms, *β* = -13.4 ms, SE = 16.133, *p* = .410), but none of these differences reached statistical significance (Fig. 5C).

## Discussion

### Confirmation of the speed-accuracy trade-off when the accuracy level of the landing in the vertical jump task is changed

This study conducted a vertical jump task under three conditions: normal conditions, in which landing position control was not required, and three levels of adjusted conditions, in which landing position control was required. The main results of this study are as follows: under all conditions, the participants performed jumps with maximum effort. However, as the accuracy of the landing position became more critical, the entropy value of the 2D position vector of the landing point decreased (improving accuracy), and the jump height decreased (reducing speed). Given that the vertical velocity at take-off determines the jump height, the velocity was adjusted to improve the accuracy of the landing point^9,26^.

In our previous study^9^we showed that the speed-accuracy trade-off observed in hand movement experiments can also occur in single discrete whole-body movements that take approximately 1 s to complete (similar to the normal condition in the present study), based on experiments in conditions where accuracy was controlled by language instruction and in conditions where it was not. This study reveals that this relationship changes further at the required level of accuracy. This suggests that the same effect as the experimental results, in which the performance measure, movement time (MT), changes according to the task difficulty ID, which changes according to the accuracy requirements led by hand movement^2,3^also occurs in whole-body movements. Specifically, even with a single discrete whole-body movement, the movement speed is a trade-off depending on the required level of endpoint accuracy. The current study expands the understanding of speed-accuracy trade-offs by demonstrating their existence in whole-body movements, such as vertical jumping. These findings bridge the gap between the research on fine motor tasks and complex athletic movements. In summary, similar to the findings of hand movement studies (e.g^2,3^). , , stricter accuracy requirements led to a reduced movement speed in vertical jumping. However, unlike fine motor tasks, whole-body movements are constrained by biomechanical factors, such as take-off power and landing stability. Notably, these trade-offs occurred despite the participants’ inability to continuously monitor the target during the jump, relying instead on the initial recognition of accuracy constraints. Additionally, the results of this study, which showed that physical constraints (such as changes in target size) can cause changes in movement accuracy, have also been shown in other studies^27,28^strengthening the suggestion that such methods can be helpful when examining other sports movements.

Furthermore, analyzing all trial data rather than participant-level averages was motivated by the need to investigate trends across trials while maintaining a practical experimental design. Analyzing participant-level averages would have limited the sample size to 12 observations per condition, reducing statistical power. Treating each trial as an independent observation supported by an experimental design that minimized intertrial dependencies allowed for a more precise assessment of the effect of landing accuracy requirements on jump performance. The robustness of the findings was confirmed by consistent results of a secondary analysis using participant-level averages.

### Mechanism behind the speed-accuracy trade-off of vertical jump

To investigate the mechanism behind the trade-off phenomenon in the vertical jump, we examined the characteristics of the vertical jump that have been highlighted in previous studies, such as the fact that the center of gravity moves in a parabolic motion in the air and the landing position is determined when the jumper leaves the ground^9^. In other words, we analyzed how the adjustment of movement from the start to the point of leaving the ground affects landing accuracy. Specifically, we examined three aspects: the velocity vector’s scalar, directional, and duration movement times.

**Fig. 6:**
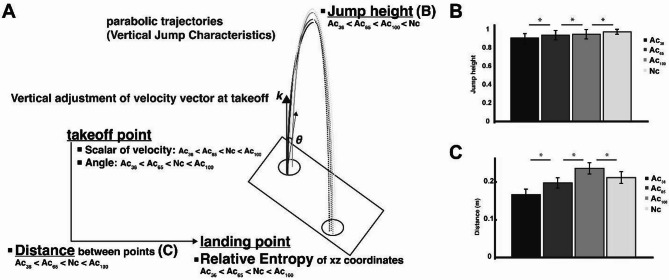
Conceptual diagram of each parameter related to position adjustment in a vertical jump. (Adapted from Fig. 5 in Murakami & Yamada (2024)). (**A**) Schematic diagram of parabolic jump trajectories and velocity vector adjustment at take-off. The scalar velocity and angle of deviation were modulated across the accuracy conditions in the order of Ac_36_ < Ac_65_ < Nc < Ac_100_ for both parameters. Jump height (**B**) and the distance between the take-off and landing points (**C**) were plotted, showing a graded change across conditions. The relative entropy at the landing point decreased with increasing accuracy demands, indicating enhanced landing precision. The error bars in the bar graphs represent standard deviations across participants. Significant differences between conditions are indicated by asterisks (*p < .05).

Figure 6 shows a conceptual diagram based on the parameters of the conditions calculated from the actual analysis that may have influenced the adjustment of the landing point during a vertical jump. The present study’s findings extend our previous research^9^which demonstrated that instructing participants to land on the take-off point during vertical jumping led to increased accuracy (as reflected by lower entropy) and reduced jump height. By examining changes in take-off speed and direction, we previously showed that accuracy was achieved by lowering the scalar velocity and adopting a more vertical trajectory. As a result, the mean scalar and mean angle of deviation from the vertical direction of the 3D velocity vector between the most vertical sink point and the take-off point decreased significantly as the accuracy level increased. The results showed that as the accuracy of the landing was adjusted, the magnitude and direction of the velocity vector acting during this time were adjusted. The entropy values calculated from the 2D position vectors of the landing points decrease as the landing accuracy requirement increases (Ac_36_: 1.99, Ac_65_: 2.40, and Ac_100_: 2.94). The information processing characteristics (reduction in the amount of information between points in time as time progressed) revealed by hand movement were adjusted during trajectory (adjusted by movements related to feedback control)^29,30^. In contrast, a previous study of the vertical jump^9^ suggested that the trajectory is essentially controlled differently, with the initial velocity direction set at take-off and the trajectory determined in advance using a feed-forward method. This study showed that such control in the vertical jump was more pronounced and required greater landing accuracy. This supported the application of entropy analysis to movement analysis, which was shown to be very effective in quantifying information processing during movement, and this control process is evaluated as “apparent” information generation during the aerial phase^9^. Notably, unlike hand movements, vertical jumps do not permit trajectory adjustments after take-off due to the ballistic nature of the motion. Once airborne, the take-off velocity and angle physically determine the trajectory, and feedback-based corrections are not possible. Therefore, the observed reduction in entropy at the landing point under high-accuracy conditions should not be interpreted as a result of information processing occurring during the aerial phase. Instead, it reflects the feed-forward control strategies implemented before take-off. In this sense, increased information processing “during” the aerial phase appears to be the consequence of anticipatory motor planning—consistent with our previous findings^9^ on feed-forward regulation in constrained whole-body movements.

Next, we examined the time taken in two sections: the time from the most vertical sink point to the take-off point and the starting movement point to the most vertical sink point. We found no significant difference in the time taken in the former section between conditions; however, the time taken in the latter section was longer in situations that required greater accuracy. These results may further strengthen the role of action movement time in improving landing accuracy, as shown in a previous study^9^. Precisely, it can be thought that this reinforces the prior idea of mechanism that the final position adjustment was made by recognizing the current direction of movement while spending time on this phase, applying force, and adjusting the speed vertically in the first half of the vertical acceleration.

While this study focused on the overall trends across trials, individual differences in motor control strategies were not explicitly analyzed. Future research could investigate these differences to provide a more comprehensive understanding of speed-accuracy trade-offs in whole-body movements.

### Interpreting the results of conditions that do not require physical accuracy: comparing self-generated condition and externally controlled accuracy condition

A different and interesting trend was observed under condition (Nc), which did not require landing accuracy. As the landing necessary accuracy increased, a trade-off occurred between jump height and landing accuracy. However, when comparing Nc with Ac_100_, Nc had a higher jump height and landing accuracy as evaluated from entropy. This result suggests that the influence of differences in conditions between self-generated and externally controlled movements, which has been widely discovered in research on tapping movements^20,31^may also be relevant to vertical jumping movements. Research on tapping movements has shown that movements performed at a self-generated (self-pacing) frequency are more comfortable than those performed at an externally generated (external pacing) frequency, resulting in different motor control styles. Similarly to the conditions with self-generated frequencies, in this study, conditions where landing accuracy was not required likely allowed participants to perform movements comfortably. This reduced the trade-off between landing accuracy and jumping height compared to the conditions where landing accuracy was externally altered. The changes in performance due to these differences in conditions need to be examined further.

### Limitations and future directions

While the study was designed to address its core research question with methodological rigor and precision, no experimental design can capture all dimensions of a complex phenomenon, such as whole-body movement. Therefore, the following limitations are noted not as shortcomings but as opportunities to refine and expand upon this line of inquiry in future work.

One limitation of this study is its relatively small sample size (*n* = 12), which, although partially addressed by using linear mixed-effects models (LMMs), may affect the generalizability of the findings. Additionally, although the participants were experienced athletes from various disciplines, the study did not focus on sports-specific performance differences. The lack of biomechanical data (e.g., joint kinetics and muscle activation) also limits our ability to infer the underlying motor control mechanisms.

Furthermore, although filtering is commonly used to remove high-frequency noise from biomechanical signals, we intentionally chose not to apply filters to the raw force data. This methodological decision was supported by frequency-domain analysis using the Fast Fourier Transform (FFT) and Power Spectral Density (PSD) performed on the vertical force signal up to the take-off point. The results (Supplementary Fig. 1) showed that the dominant frequency components were within the expected low-frequency range (predominantly below 10 Hz), and the spectral power above 20 Hz was negligible across all axes. These findings suggest that the contribution of high-frequency noise to the raw signal was minimal. This approach is consistent with previous studies’ recommendations that emphasize preserving signals’ natural characteristics when applying entropy-based and complexity measures to movement data^32,33^. In our case, filters may have unintentionally removed meaningful signal variability, essential for entropy analysis. Nevertheless, the decision to forgo filtering may not be optimal in all contexts.

We believe presenting unfiltered data supported by an objective frequency analysis offers a transparent and empirically justified rationale for our methodological choice. However, we recognize this as a point of discussion and a potential direction for methodological refinement in future studies.

## Conclusion

This study investigated the speed-accuracy trade-off in vertical jump tasks by systematically varying the landing accuracy requirements. The findings demonstrated that stricter accuracy demands reduced jump height and take-off velocity, confirming a clear trade-off between speed and accuracy across the three levels of landing precision in whole-body movements. Entropy analysis revealed decreased variability in the landing positions under higher-accuracy constraints, providing evidence for precision adjustments in motor control. Notably, these trade-offs emerged even though the participants could not continuously monitor the landing target during the jump, relying instead on the initial recognition of target constraints before take-off. Adjustments in the magnitude and direction of the take-off velocity and prolonged movement preparation times highlight the role of feed-forward control in meeting accuracy demands.

While the observed trade-offs align conceptually with Fitts’ law, they are specific to the experimental conditions and have not been formalized into a mathematical framework. Rather than extending Fitts’ law directly to whole-body movements, these findings suggest that similar principles may govern speed-accuracy relationships in gross motor tasks. This study advances our understanding of dynamic full-body movement by clarifying the interplay among jump height, accuracy constraints, and motor control strategies. Furthermore, the results highlighted the ability of the motor system to adjust precision based on the initial recognition of task constraints, even without continuous visual feedback. These findings have potential applications in sports science, robotics, rehabilitation, and movement optimization technologies. Future research should aim to develop formal models of these trade-offs and investigate their generalizability to other dynamic tasks.

### Competing interests

The authors declare no competing interests.

## Electronic supplementary material

Below is the link to the electronic supplementary material.

Supplementary Material 1

Supplementary Material 2

## Data Availability

The datasets and programming codes generated or analyzed in the current study are available from the corresponding author upon reasonable request.
