# Supplementary material for: Speed and accuracy tradeoff in whole body movement during vertical jumps under varying landing constraints
Source: Sci Rep. 2025 Jun 6;15:19966. doi: 10.1038/s41598-025-04601-4 (PMC12144266; doi:10.1038/s41598-025-04601-4)
Supplement: Supplementary file 2 — Supplementary Material 2 [file 41598_2025_4601_MOESM2_ESM.docx]

**Supplementary Figure 1**: Frequency analysis of the unfiltered force data from the start of the movement to the take-off phase

A) Raw force time series data (x, y, and z axes). B) Power Spectral Density (PSD) plots on a linear scale (N²/Hz) for Fx, Fy, and Fz computed from the FFT of unfiltered force data. Across all axes, the primary spectral components were concentrated below 10 Hz, and the power declined rapidly beyond 20 Hz, indicating minimal high-frequency noise in the force data.
